# Ultrasound-mediated synthesis of *N*,*N*-bis(phenacyl)aniline under solvent-free conditions

**DOI:** 10.1007/s00706-013-0978-7

**Published:** 2013-04-26

**Authors:** Jingyu He, Lanxiang Shi, Sijie Liu, Peng Jia, Juan Wang, Ruisheng Hu

**Affiliations:** College of Chemical Engineering, Shijiazhuang University, Shijiazhuang, 050035 People’s Republic of China

**Keywords:** *N,N*-bis(phenacyl)aniline, Ultrasound irradiation, Solvent free synthesis

## Abstract

**Abstract:**

An efficient method for the synthesis of *N*,*N*-bis(phenacyl)anilines was developed via smooth condensation of anilines with α-bromoacetophenones in the presence of sodium carbonate as acid acceptor and polyethylene glycol 400 (PEG 400) as catalyst at room temperature under solvent-free conditions by using 350 W ultrasound irradiation. In addition to experimental simplicity, the main advantages of the procedure are mild conditions, short reaction times (30–45 min), and high yields (73–83 %).

**Graphical Abstract:**

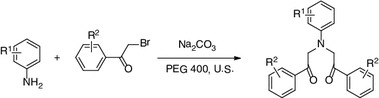

## Introduction

*N*,*N*-Bis(phenacyl)anilines are of particular importance in the fine chemical industry owing to their applications as precursors of various heterocyclic compounds such as piperidine, triazepine, 1,4-dihydropyrazine, and indole [1–4]. Traditional methods for the synthesis of *N*,*N*-bis(phenacyl)aniline were achieved through the alkylation of anilines with α-bromoacetophenone, which gave low and unsatisfactory yields, and needed organic solvents and long reaction times [5–7]. In addition, the grinding method was also used in the preparation of *N*,*N*-bis(phenacyl)aniline; however, the method applied only to small-scale production, which is its main disadvantage [2].

**Scheme 1:**
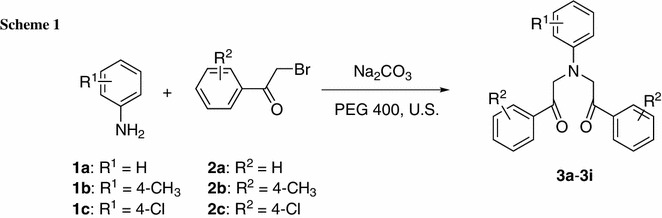
.

Phase transfer catalysis (PTC) is widely applied in many industries such as fine chemicals, agrochemicals, specialty chemicals, pharmaceuticals, perfumes, flavors, dyes, and polymers, and even pollution and environmental control processes [8–10]. PEGs are soluble, recoverable, thermally stable, and inexpensive phase transfer catalysts. PEG and its many derivatives have become popular and are used in several commercial processes to replace expensive and environmentally harmful phase transfer catalysts in PTC reactions [11–13]. Recently, ultrasound-assisted solvent-free reactions have emerged as valuable tools in organic synthesis that use less organic solvent, milder conditions, shorter reaction times, and afford excellent yields with higher selectivity [14–17]. PTC and ultrasound are two clean and useful protocols in organic synthesis; ultrasonic irradiated synthesis of some interesting heterocyclic compounds under PTC has been listed in several reports [18–21]. In the framework of our investigations on the development of green chemical procedures [22, 23], we herein report a novel and environmentally safe procedure for the rapid preparation of *N*,*N*-bis(phenacyl)anilines in the presence of sodium carbonate as acid acceptor and PEG 400 as catalyst at room temperature without solvent under ultrasound irradiation (Scheme 1). This approach is designed to overcome the limitations previously encountered in the reaction.

## Results and discussion

For initial optimization of the reaction conditions, a mixture of aniline (**1a**), α-bromoacetophenone (**2a**), and sodium carbonate was irradiated in the presence of PEG 400 at room temperature (Scheme 1). By increasing the irradiation power from 200 to 400 W, the reaction time of **3a** decreased from 2.5 h to 50 min and the yield increased from 38 to 54 %. The data in Table 1 show that the reaction time and yield of **3a** did not change when the power changed from 350 to 400 W; therefore, 350 W of ultrasonic irradiation was sufficient to promote the reaction. The best yield for **3a** was obtained by ultrasonic irradiation for 50 min at room temperature and 350 W in the presence 2 mol% PEG 400. It is therefore obvious that ultrasound irradiation accelerates the condensation of aniline with α-bromoacetophenone possibly owing to the simultaneous heating and transport caused by ultrasound [24].

**Table 1:** Yields of **3** under ultrasound solvent-free conditions

Entry	Power/W	Time/min	Yield^a^/%
1	200	150	38
2	250	120	41
3	300	80	46
4	350	50	54
5	400	50	54

Aniline (10 mmol), α-bromoacetophenone (20 mmol), sodium carbonate (10 mmol), and 2 mol% PEG 400

^a^Isolated yield

The catalytic activity of PEG 400 was also studied. A blank reaction was conducted with aniline, α-bromoacetophenone, and sodium carbonate in the absence of catalyst, and *N*,*N*-bis(phenacyl)aniline **3a** was obtained in 11 % yield after 2.5 h. With increasing amounts of PEG 400 (1, 2, 3, 4, 5, 6 mol%), **3a** was produced in 35 to 82 % and the reaction time decreased from 90 to 30 min (Table 2). The use of 5 mol% of PEG 400 was sufficient to promote the reaction, whereas a larger amount of the catalyst did not improve the results greatly. Therefore, 5 mol% of PEG 400 was chosen as the optimal catalyst amount for the synthesis of **3a**.

**Table 2:** Effect of the amount of PEG 400 on the synthesis of **3a** under ultrasound irradiation

Entry	PEG 400/mol%	Time/min	Yield^a^/%
1	0	150	11
2	1	90	35
3	2	80	54
4	3	60	71
5	4	50	77
6	5	30	81
7	6	30	82

Aniline (10 mmol), α-bromoacetophenone (20 mmol), sodium carbonate (10 mmol), and 350 W ultrasound

^a^Isolated yield

The reactions generally gave high yields of products (Table 3). No obvious electronic effect of the substituents of the anilines was observed in the reactions because electron-donating as well as electron-withdrawing groups were well tolerated. Compound **3d** was previously prepared in low and unsatisfactory yield in EtOH as solvent [5], and **3b** in 64 % yield by the grinding method [2], whereas our procedure gave these *N*,*N*-bis(phenacyl)anilines in 79 and 83 % yield, respectively. Compounds **3c** and **3f** were previously prepared in 70 and 68 % yield, respectively, by grinding method after 1 h [2]. Our procedure gave **3c** and **3f** in 77 and 81 % yield, respectively, within 40 min.

**Table 3:** Synthesis of *N*,*N*-bis(phenacyl)anilines **3** under ultrasound conditions

	R^1^	R^2^	Time/min	Yield^a^/%		M.p./°C	
				Found^b^	Reported	Found^c^	Reported
**3a**	H	H	30	81	9 [5]	235.7–236.5	236–240 [5]
**3b**	H	4-Me	30	83	64 [2]	102.5–103.3	103 [2]
**3c**	H	4-Cl	40	77	70 [2]	111.4–112.6	110 [2]
**3d**	4-Me	H	30	79	– [3]	254.2–255.3	255 [6]
**3e**	4-Me	4-Me	40	80	70 [2]	127.6–128.9	128 [2]
**3f**	4-Me	4-Cl	30	81	68 [2]	141.7–143.0	140 [2]
**3g**	4-Cl	H	30	75	–	158.3–160.2	[25]
**3h**	4-Cl	4-Me	40	80	–	178.5–181.3	–
**3i**	4-Cl	4-Cl	50	73	–	181.5–182.7	–

All the isolated products were characterized by their physical properties, by ^1^H NMR and IR spectra, and by direct comparison with literature data [2, 5, 6]

^a^Yield of isolated product

^b^This work

In summary, we developed an efficient method for the synthesis of bis(phenacyl)anilines via condensation of anilines with α-bromoacetophenones in the presence of sodium carbonate as acid acceptor and PEG 400 as catalyst at room temperature under solvent-free conditions by using 350 W ultrasound irradiation.

## Experimental

All chemicals and reagents were purchased from commercial sources and used without further purification. Melting points were determined on an X-5 instrument. IR spectra were performed as KBr pellets on a Bruker VERTEX 70 spectrophotometer. NMR spectra were measured on a Bruker AVANCE 400 spectrometer. MS spectra were recorded on a ZAB-HS and ESQUIRE 6000 mass spectrometer. Ultrasonication was performed in a GEX750-5C ultrasonic processor equipped with a 3-mm-wide and 140-mm-long titanium alloy probe that was immersed directly into the reaction mixture. In all reactions the tip of the sonotrode was located in the same position just under the liquid surface in order to obtain optimal sonication and reproducible results. The operating frequency was 24 kHz and the output power was 0–750 W through manual adjustment. The reactions were carried out in a four-neck pear-shaped flask of 50 cm^3^ capacity in the open air. The temperature was controlled by a Büchi B-491 water bath at room temperature (25 ± 1 °C). Elemental analyses were carried out on a Carlo Erba 1106 elemental analysis instrument.

### General procedure for the preparation of the *N,N-*bis(phenacyl)anilines **3a**–**3i**

A mixture of the aniline (10.0 mmol), α-bromoacetophenone (20 mmol), sodium carbonate (10.0 mmol), and 0.2 g PEG 400 (0.5 mmol) was irradiated under an ultrasonic processor at room temperature and 350 W in the open air. The reactions were completed within 30–45 min. The product was washed or recrystallized from 15 cm^3^ EtOH to afford the *N*,*N*-bis(phenacyl)aniline. All products were identified by their melting points, IR, ^1^H NMR spectra, and ^13^C NMR spectra and comparison with reported data [2, 5] (see also Table 3).

#### *N,N*-*Bis(4*-*methylphenacyl)*-*p*-*chloroaniline* (**3h**, C_24_H_22_ClNO_2_)

Light yellow crystals; M.p.: 178.5–181.3 °C; IR (KBr): $\bar{v}$ = 3,033, 1,663, 1,510, 1,479, 1,375, 1,112, 792 cm^−1^; ^1^H NMR (400 MHz, CDCl_3_): *δ* = 2.35 (s, 6H, CH_3_), 4.92 (s, 4H, N-CH_2_), 6.45 (d, *J* = 9.2 Hz, 2H, Ar–H), 7.09–7.11 (m, 2H, Ar–H), 7.49–7.53 (m, 4H Ar–H), 7.61–7.65 (m, 2H, Ar–H), 8.00–8.02 (m, 4H Ar–H) ppm; ^13^C NMR (100 MHz, CDCl_3_): *δ* = 58.3, 113.5, 120.3, 128.2, 128.9, 130.4, 135.6, 141.2, 149.5, 195.8 ppm; MS: *m*/*z* = 391 (M^+^).

#### *N,N*-*Bis(4*-*chlorophenacyl)*-*p*-*chloroaniline* (**3i**, C_22_H_16_Cl_3_NO_2_)

Light yellow crystals; M.p.: 181.5–182.7 °C; IR (KBr): $\bar{v}$ = 3,033, 1,663, 1,510, 1,479, 1,375, 1,112, 792 cm^−1^; ^1^H NMR (400 MHz, CDCl_3_): *δ* = 4.95 (s, 4H, N-CH_2_), 6.51 (d, *J* = 8.8 Hz, 2H, Ar–H), 7.08–7.10 (m, 2H, Ar–H), 7.43–7.58 (m, 4H Ar–H), 7.61–7.72 (m, 2H, Ar–H), 8.00–8.08 (m, 4H Ar–H) ppm; ^13^C NMR (100 MHz, CDCl_3_): *δ* = 58.3, 113.5, 120.3, 128.2, 128.9, 130.4, 135.6, 141.2, 149.5, 195.8 ppm; MS: *m*/*z* = 431 (M^+^).
